# Diagnosis of severe community-acquired pneumonia caused by *Acinetobacter baumannii* through next-generation sequencing: a case report

**DOI:** 10.1186/s12879-019-4733-5

**Published:** 2020-01-15

**Authors:** Ancong Xu, Hong Zhu, Bingqi Gao, Haixu Weng, Zhangna Ding, Mianmian Li, Xing Weng, Guoxin He

**Affiliations:** 1 The Third Affiliated Hospital of Wenzhou Medical University, 108 Wansong Road, Wenzhou, 325200 Zhejiang China; 2 BGI-Shenzhen, Shenzhen, 518083 Guandong China

**Keywords:** *Acinetobacter baumannii*, Next generation sequencing, Community-acquired pneumonia

## Abstract

**Background:**

*Acinetobacter baumannii* is a gram-negative aerobic bacillus that is commonly causes of hospital-acquired infections. Community-acquired pneumonia caused by *Acinetobacter baumannii* (CAP-Ab) is rare but fatal if diagnosis and treatment are delayed. Conventional culture of clinical specimens is the main method for clinical diagnosis of *A. baumannii* infections which may suffer from limited positive rate and is time consuming. Timely and precise diagnosis of CAP-Ab remains challenging.

**Case presentation:**

A 66-year-old man with 24 h history of acute fever and dyspnea was admitted to our hospital. He was diagnosed as severe community acquired pneumonia (CAP), septic shock, respiratory failure and acute kidney injury. Next-generation sequencing (NGS) was performed on the patient’s sputum and blood, which identified numerous *A. baumannii* nucleotide sequences in the sample of sputum and led to the rapid diagnosis and treatment of community acquired pneumonia caused by *A. baumannii*. This result was confirmed by subsequent sputum culture.

**Conclusions:**

This case described that the successful application of the next generation sequencing assisting the speedy diagnosis of *A. baumannii* infection provides a new idea for the timely diagnosis of CAP-Ab and highlights that NGS is a promising tool in rapid etiological diagnosis of acute and severe infectious diseases.

## Background

*Acinetobacter baumannii* is a gram-negative aerobic bacillus that is usually found in fresh water and soil [1]. It mainly causes hospital-acquired pneumonia (HAP), especially those with low immunity, long-term hospitalization and use of broad-spectrum antibiotics [2, 3]. *A. baumannii* has innate resistance mechanisms against multiple antimicrobials on its core genome, which is also one of the reasons for its tendency to cause outbreaks [4]. Community-acquired pneumonia caused by *A*. *baumannii* (CAP-Ab) is rare. However, CAP-Ab progresses more rapidly and is associated with a higher mortality compared to HAP caused by *A*. *baumannii* [4]. Therefore, early diagnosis and appropriate antimicrobial therapy are the key to improve the prognosis of CAP-Ab. Here we report a case of a middle-aged Chinese man presenting with acute fever and dyspnea, diagnosed as severe community-acquired pneumonia. In this case, we used next-generation sequencing (NGS) to expeditiously identify *A*. *baumannii* as the causative agent from the sputum of patient which provided a valuable direction for early clinical diagnosis and guided the therapy. To our knowledge,this is the first case of NGS assisting in the diagnosis of CAP-Ab and highlighting the potential of such technique in the rapid etiological diagnosis of acute and severe infectious diseases in the future.

## Case presentation

A 66-year-old man presented to the emergency department of the Third Affiliated Hospital,Wenzhou Medical University (Wenzhou, China) with acute fever for 24 h. He also complained of intermittent productive cough with pale bloody sputum associated with chest heaviness, pectoralgia and dyspnea at rest. The patient was previously healthy and had no definite history of epidemiological contact except for there was a history of poultry contact 3 days ago. However, he said that he had a 50-year history of cigarette smoking and alcohol ingestion and had not quit before being sent to our hospital. He was not currently employed. Besides, he had no previous hospitalization, antimicrobial use history and healthcare institution visiting record in 1 year. Furthermore, he had never sought medical treatment for his current condition.

On admission, he appeared acutely ill, but was conscious. His blood pressure was 100/69 mmHg, respiratory rate 26 breaths per minute, pulse rate 119 beats per minute, and was febrile with body temperature of 38.8 °C. A regular heart rhythm was observed. There were coarse rales in the right lung field. Under 5 L/min oxygen flow through nasal catheter, the oxygen saturation can only be maintained at 85–90%.

**Fig. 1:**
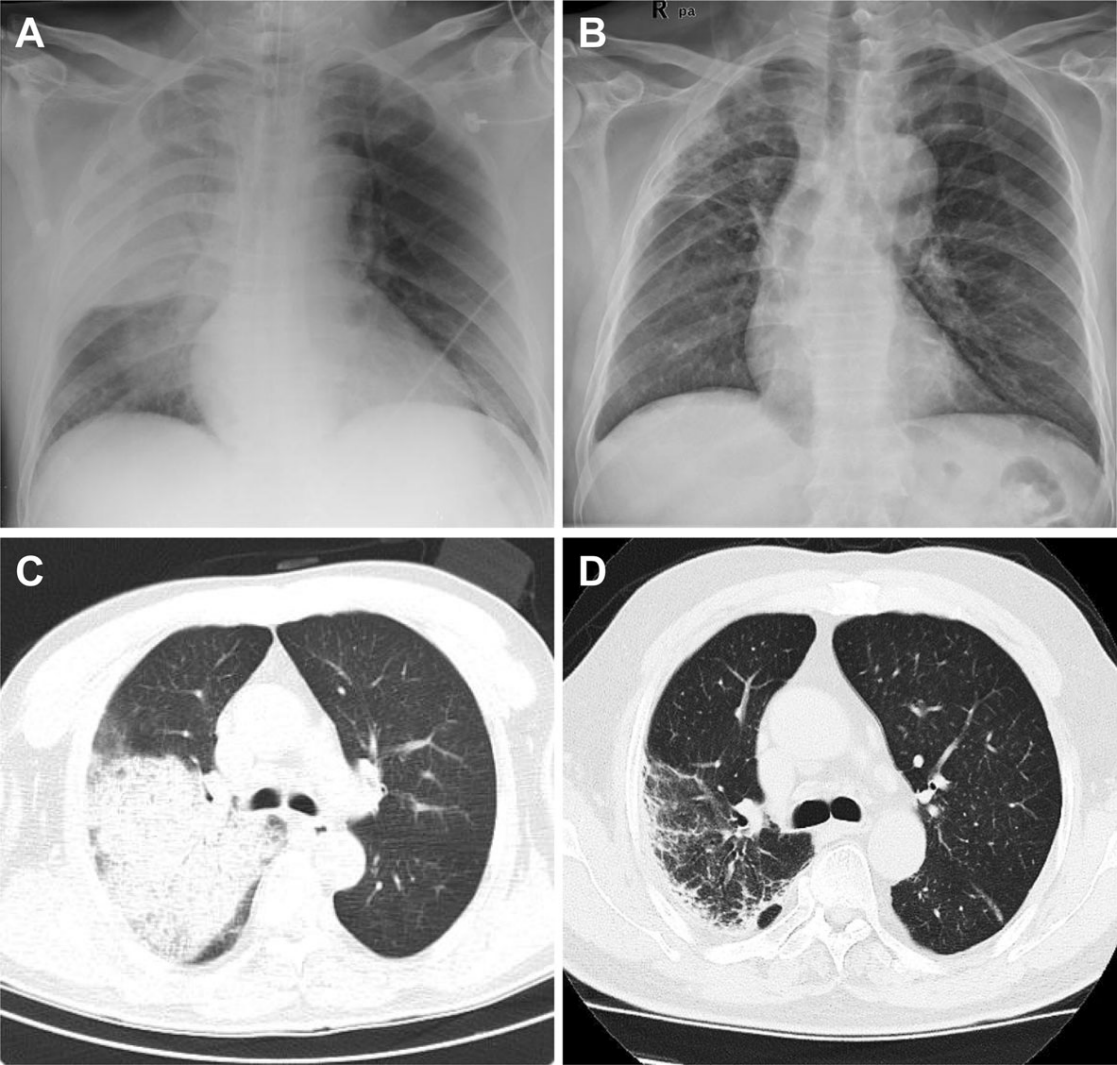
Chest X-ray and computed tomography on admission and the 28th day after admission. Chest X-ray on admission showed infiltrative shadow from the right upper, middle and lower lung lobes (**a**). Computed tomography on admission showed multiple exudate consolidation in the right upper, middle and lower lung lobes (**c**). On the 28th day after admission, the chest X-ray (**b**) and computed tomography (**d**) showed improvement in the consolidations of the right upper, middle and lower lung lobes

The laboratory results were as follows: white blood cell count 5.0 × 10^9^/L with neutrophil predominance (72.0%), hemoglobin 163 g/L, platelet count 181 × 10^9^/L, and inflammatory markers was significantly increased, C-reactive protein (CRP) 133.68 mg/L, procalcitonin (PCT) 18.98 ng/ml. Creatinine 177 μmol/L, alanine aminotransferase 24 U/L, total bilirubin 33.1 μmol/L, total protein 60.1 g/L, and albumin 36.8 g/L. The arterial blood gases (O_2_ 5 L/min via nasal catheter) showed pH 7.41, pCO_2_ 28.3 mmHg, pO_2_ 62.7 mmHg, HCO_3_^−^ 17.6 mmol/L, lactate 3.6 mmol/L, and O_2_ saturation 89.4% (Table 1). A chest radiograph showed infiltration of the right upper, middle and lower lung lobes (Fig. 1a), and chest computed tomography (CT) showed multiple exudate consolidation in the right upper, middle and lower lung lobes (Fig. 1c).

**Table 1:** Laboratory data and vital signs on admission

Complete blood count and Biochemistry		Arterial blood gas (O_2_ 5L nasal catheter)	
WBC	5.0 × 10^9^/L	pH	7.41
Neu	72.0%	pO_2_	62.7 mmHg
RBC	4.88 × 10^9^/L	pCO_2_	28.3 mmHg
Hb	163 g/L	HCO_3_^−^	17.6 mmol/L
MCV	93.6 fL	Lactate	3.6 mmol/L
Plt	181 × 10^9^/L	SaO_2_	92%
PT	11.7 s		
APTT	26.5 s		
D-dimer	0.51 μg/L	Vital signs	
TP	60.1 g/L	Blood pressure	100/69 mmHg
Alb	36.8 g/L	Respiratory rate	26/min
LD	209 U/L	Pulse rate	119 beats/min
AST	20 U/L	Heart rate	119 beats/min
ALT	24 U/L	Body temperature	38.8 °C
T. Bil	33.1 μmol/L	Percutaneous oxygen saturation	90%
Cre	177 μmol/L		
Na	138.3 mmol/L		
K	3.88 mmol/L		
Cl	107.9 mmol/L		
CRP	133.68 mg/L		
PCT	18.98 ng/ml		
Glucose	6.67 mmol/L		

*WBC* white blood cell count, Neu: neutrophils, *RBC* red blood cell count, *Hb* hemoglobin, *MCV* mean corpuscular volume, *Plt* platelet, *PT* prothrombin time, *APTT* activated partial thromboplastin time, *TP* total protein, *Alb* albumin, *LD* lactate dehydrogenase, *AST* aspartate aminotransferase, *ALT* alanine aminotransferase, *T.Bil* total bilirubin, *Cre* creatinine, *CRP* C reacting protein, *PCT* procalcitonin.

A diagnosis of severe community acquired pneumonia with acute kidney injury was entertained. Intravenous ciprofloxacin were administered empirically. Four hours after admission, his respiratory distress worsened, consciousness began to blur, blood pressure dropped, and the blood oxygen saturation can only maintain in 80–85% even if he was treated with oxygen at 7 L/min via mask.

After an exigent consultation, he was transferred to intensive care unit (ICU) for septic shock and persistent type 1 respiratory failure. Emergency tracheal intubation with mechanical ventilation was administered (Fig. 3a). Fluid resuscitation therapy and vasoactive agent were used to maintain vital organ perfusion. At the same time, sputum and blood samples were sent for culture and NGS (BGI sequencing center, Wuhan, China). According to the laboratory results, Chest computed tomography and the rapid deterioration of the patient, hypervirulent *Klebsiella pneumoniae* was suspected. His antibiotics were upgraded to intravenous imipenem (500 mg Q6H) combined with moxifloxacin (400 mg daily). Considering his history of poultry contact, oral antiviral oseltamivir (150 mg Q12H) was added empirically for possibility of Influenza A virus.

**Fig. 2:**
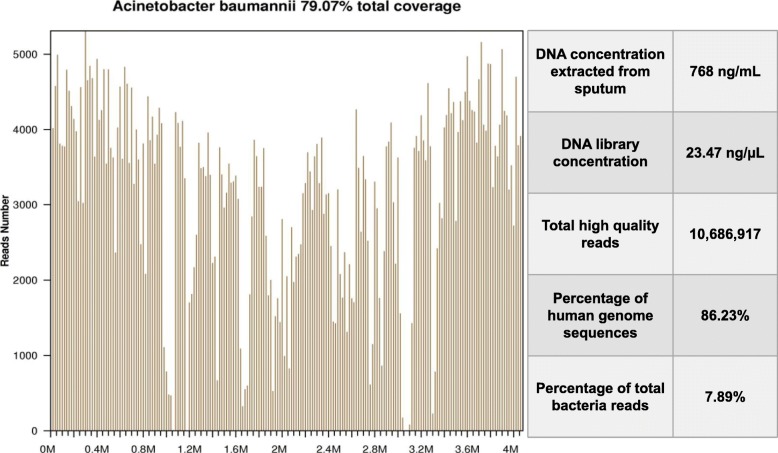
Sequence reads mapped to *A. baumannii* by mNGS data. A total of 274,127 reads mapped to *A. baumannii* in the reference database, which contains about 8000 pathogen genomes, corresponding to a total coverage of 79.07%

**Fig. 3:**
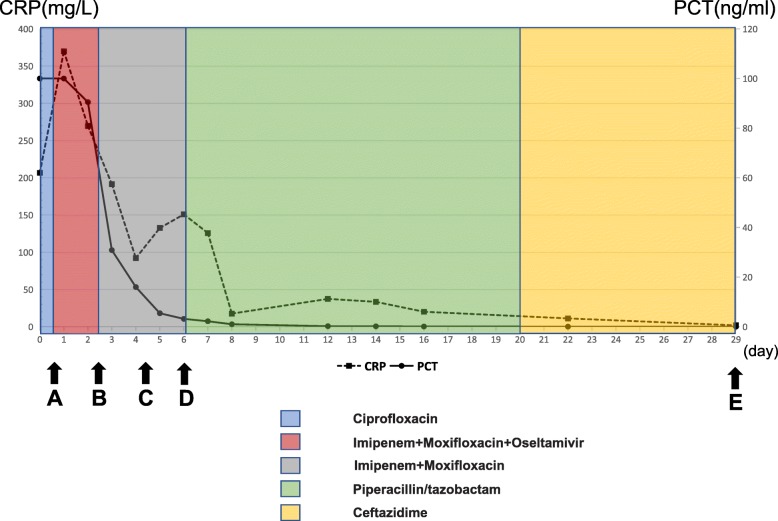
Clinical course. **a** Emergency tracheal intubation with mechanical ventilation was administered. At the same time, sputum and blood samples were sent for culture and NGS. **b** *A. baumannii* was identified in the sputum samples by NGS, but no specific pathogens complex nucleotide sequences were detected in the sample of blood. **c** *A. baumannii* was also identified in the culture of sputum samples, and the cultures of blood came back negative as well. **d** The patient was extubated. **e** The patient was discharged. CRP: C reacting protein, PCT: procalcitonin

NGS detection was completed within 48 h (Fig. 3b). Its procedure and results were as follows: a volume of 300 uL plasma and sputum samples were taken for RNA extraction using the QIAamp Viral RNA Mini Kit (QIAGEN, Germany) following the manufacturer’s instruction. Then complementary DNA (cDNA) was generated from the extracted RNA templates by reverse transcription. Specimens were used for the construction of DNA libraries following published procedure, including DNA-fragmentation, end-repair, adapter-ligation and PCR amplification and quality control by Agilent 2100. Qualified libraries were sequenced by BGISEQ-50 platform. After removing low-quality and short (< 35 bp) reads, quality-passed sequencing were processed for computational substraction of human host sequences (hg19) and alignment to updated Microbial Genome Databases, consisting of 1798 viruses, 6350 bacteria, 1064 fungi, and 234 parasites. The classification reference databases were downloaded from NCBI (ftp://ftp.ncbi.nlm.nih.gov/genomes/). NGS detected 274,127 of 20 million reads that matched *A. baumannii* from the sputum samples (Fig. 2).

For the analysis of antibiotic resistant genes, the CARD included analyzing software Resistance Gene Identifier (RGI) was used to predicts antibiotic resistance genes from the metagenomic sequence data and the un-annotated genome sequence assembly contigs (Additional file 1: Table S1 and S2).

According to the results of NGS and clinical manifestation of the patient, we judged that *A. baumannii* was the main pathogen, and this *A. baumannii* strain was susceptible to imipenem which was confirmed by subsequent antibiotic susceptibility results (Table 2). After ruling out the possibility of viral infection, we discontinued oseltamivir. Two days after the NGS results were reported, *A. baumannii* was also identified in the culture of sputum samples taken on admission (Fig. 3c). No specific pathogens complex nucleotide sequences were detected in the sample of blood by NGS, and the cultures of blood came back negative as well. The antibiotic susceptibility of the *A. baumannii* isolate were tested by using the broth microdilution method. The results showed the strain was susceptible to ampicillin/sulbactam, piperacillin/tazobactam, ceftazidime, cefepime, imipenem, amikacin, gentamicin, tobramycin, ciprofloxacin, levofloxacin, sulfamethoxazole and cefoperazone/sulbactam (Table 2).

**Table 2:** The antibiotic susceptibility of the *Acinetobacter baumannii* isolate

Antibiotic	Susceptibility
Ampicillin/sulbactam	S
Piperacillin/tazobactam	S
Ceftazidime	S
Cefepime	S
Imipenem	S
Amikacin	S
Gentamicin	S
Tobramycin	S
Ciprofloxacin	S
Levofloxacin	S
Sulfamethoxazole	S
Cefoperazone/sulbactam	S
Ceftriaxone	I

*S* susceptible, *I* intermediate resistance

Five days after admission, patient’s vital signs stabilized, and the fever subsided. Follow-up CRP and PCT showed improved consolidation (Fig. 3). Six days after admission, the patient was extubated and set up for nasal high-flow therapy and changed imipenem to intravenous piperacillin/tazobactam (4.5 g Q8H) based on culture susceptibility (Fig. 3d). Seven days after admission, he was transferred out of ICU. Thereafter, the patients’ respiratory condition gradually improved. Twenty days after admission, piperacillin/tazobactam was changed to intravenous ceftazidime (1 g Q8H) as further antibiotic de-escalation therapy. Twenty-eight days after admission, the chest radiograph (Fig. 1b) and chest CT (Fig. 1d) showed that the pneumonia had improved. The patient was discharged on day 29 and followed as an outpatient with no significant complications (Fig. 3e).

## Discussion and conclusions

We presented a case of CAP-Ab in a Chinese patient that was successfully treated. As far as we know, this is the first case report describing the timely diagnosis of community-acquired *A. baumannii* pneumonia with the assistance of NGS, which revealed *A. baumannii* as the causative agent from sputum samples taken on admission, leading to a prompt treatment and rapidly mitigate the disease. In this case, NGS yielded supportive information which was in consistent with the patient’s clinical features and sputum culture result that was useful in the early diagnosis of CAP-Ab.

*A. baumannii* is a rare but serious cause of community-acquired infection that was mainly reported from countries in tropical or subtropical [5, 6]. But in recent years there have been reported cases in North America as well [7]. It is more commonly reported as a pathogen of HAP than CAP. However, the latter has a higher mortality rate and progressed more rapidly than the former [2, 3]. Some risk factors for developing CAP-Ab infection have been identified, such as: smoking, alcoholism, diabetes mellitus, malignancies, renal disease and chronic lung disease [8, 9]. In our case the patient had no co-morbidities but was a smoker and alcoholic.

CAP due to *A. baumannii* characterized by a sudden onset of fever, respiratory distress, septic shock, multiorgan failure and possible death. Common symptoms include productive cough with purulent or blood-stained, shortness of breath, drowsiness or pleuritic chest pain [10]. The mortality rate of CAP-Ab is high (40–64%) [11], but, it’s reported that if early effective antibiotics against *A. baumannii* were used, the mortality rate can be reduced to only 11% [12]. Therefore, early identification of pathogens is crucial links during the treat of CAP-Ab. However, due to the rarity of CAP-Ab and the difficulty for the traditional clinical microbial diagnostic methods to identify the pathogen timely, it is hard for clinicians to consider *A. baumannii* as the pathogen of severe community acquired pneumonia at the first time [13]. This can lead to inappropriate treatment and poor prognosis.

The gold standard for diagnosis of infections relies mainly on the isolation of pathogens. Conventional culturing of clinical samples is the most common used method, nevertheless, it is a time-consuming process that always takes several days or even weeks, which might delay the most optimal treatment times [14]. Previous study reported that Gram staining can quickly identify etiologic agents in the management of CAP-Ab, however, its usefulness in the initial management of CAP remains controversial [6]. In recent years, NGS as an emerging method began to be applied in clinical field. Since the publication of the first report on the application of NGS in the diagnosis of infectious diseases in 2014, more and more studies on the rapid detection of pathogens by NGS have been published [15–18]. NGS compares the detected nucleotides from the targeted samples against the catalogue library of clinical pathogens to ascertain the possible causative agent in the clinical samples [18]. Currently, Nucleic acid amplification tests (NAATs) methods for the rapid detection of major pathogens responsible for serious morbid conditions are commercially available in microbiological laboratory diagnostics. Nevertheless, the commercial NAATs requires a hypothesis of possible pathogen before certain primers or kits are chosen and the tests are conducted. While an accurate guess on the pathogenic microbes is often challenging, it may delay the diagnosis and therapy. However, compared with NAATs and conventional clinical microbiology, the unbiased metagenomic NGS analysis is able to detect thousands of pathogens at a time, relieving the clinician from the pain of making a guess. That were our reasons to choose NGS as a pathogen detection method.

Under such circumstances, the implementation of NGS in clinical field has great guiding value for the diagnosis and treatment of infectious diseases. In our case, as there were no co-morbidities in the patient and the morbidity of CAP-Ab is extremely low in our region, *A. baumannii* was never considered at the outset until the NGS test results returned, providing a valuable direction for the adjustment of subsequent antibiotic prescription.

NGS tends to detect all nucleotide sequences not only from the samples but also those acquired from the contamination, thus, to ascertain whether detected microorganism sequences are the possible etiological hypothesis and whether the result is of clinical value is a major challenge of NGS in clinical practice [18]. It suggests that the sequencing results should be strictly interpreted based on the clinical manifestation of the patient and combined with other conventional clinical microbiology results.

Here, we provided a valuable case of CAP-Ab that was early and accurate diagnosed with the assistance of NGS and was successfully treated with the initial administration of carbapenem. This study highlights that NGS may greatly accelerate the identification of diseases caused by uncommon pathogens through complementing the traditional clinical microbial diagnostic methods and further assist in clinical decision making.

## Supplementary information

**Additional file 1.** The molecular resistance profile of A. baumanni strain. **Table S1.** Molecular antibiotic resistance profile of the *A.baumanni* sequence assembly. **Table S2.** Molecular antibiotic resistance profile of the *A.baumanni* metagenomic sequences.

## Data Availability

All data generated or analysed during this study are included in this published article.

## Abbreviations

Alb: Albumin
ALT: Alanine aminotransferase
APTT: Activated partial thromboplastin time
AST: Aspartate aminotransferase
CAP: Community acquired pneumonia
CAP-Ab: Community acquired pneumonia Caused by *Acinetobacter baumannii*
Cre: Creatinine
CRP: C reacting protein
Hb: Hemoglobin
ICU: Intensive care unit
LD: Lactate dehydrogenase
MCV: Mean corpuscular volume
Neu: Neutrophils
NGS: Next-generation sequencing
PCT: Procalcitonin
Plt: Platelet
PT: Prothrombin time
RBC: Red blood cell count
T.Bil: Total bilirubin
TP: Total protein
WBC: White blood cell count,
