# Supplementary material for: Diagnosis of severe community-acquired pneumonia caused by Acinetobacter baumannii through next-generation sequencing: a case report
Source: BMC Infect Dis. 2020 Jan 15;20:45. doi: 10.1186/s12879-019-4733-5 (PMC6964051; doi:10.1186/s12879-019-4733-5)
Supplement: Supplementary file 1 — Additional file 1. The molecular resistance profile of A. baumanni strain. Table S1. Molecular antibiotic resistance profile of the A.baumanni sequence assembly. Table S2. Molecular antibiotic resistance profile of the A.baumanni metagenomic sequences. [file 12879_2019_4733_MOESM1_ESM.docx]

**Supplement 1:**  The molecular resistance profile of A. baumanni strain

**Table 1**. Molecular antibiotic resistance profile of the *A.baumanni* sequence assembly

| **Antibiotic resistant gene** | **Mapped gene size** | **Avg depth** | **Coverage(%)** |
| --- | --- | --- | --- |
| adeF | 1219 | 1.02 | 88.75 |
| adeS | 1086 | 0.99 | 93.36 |
| OXA-380 | 826 | 0.55 | 51.76 |
| adeR | 745 | 0.64 | 59.54 |
| adeH | 1453 | 1.04 | 90.01 |
| abeS | 331 | 0.63 | 62.73 |
| adeJ | 3178 | 1.06 | 98.21 |
| cat | 688 | 0.07 | 6.7 |
| adeB | 3109 | 0.97 | 87.64 |
| OXA-383 | 826 | 0.49 | 48.73 |
| adeG | 3181 | 0.72 | 68.77 |
| abeM | 1348 | 0.69 | 65.18 |
| adeI | 1252 | 1 | 88.09 |
| adeK | 1456 | 0.93 | 83.64 |
| tetM | 1921 | 0.03 | 2.86 |
| adeN | 654 | 0.96 | 80.55 |
| mfd | 3447 | 0.27 | 26.76 |
| ADC-2 | 1152 | 0.86 | 80.8 |
| adeL | 1014 | 1.02 | 90.62 |
| Staphylococcus aureus rpoB mutants conferring resistance to rifampicin | 3553 | 0.05 | 5.46 |
| Escherichia coli rpoB mutants conferring resistance to rifampicin | 4030 | 0.12 | 12.09 |
| Staphylococcus aureus rpoC conferring resistance to daptomycin | 3625 | 0.14 | 13.02 |
| Escherichia coli EF-Tu mutants conferring resistance to kirromycin | 1185 | 0.28 | 27.53 |
| LpxA | 789 | 1.01 | 89.85 |
| LpxC | 903 | 0.76 | 74.61 |
| Haemophilus parainfluenzae gyrA conferring resistance to fluoroquinolones | 2646 | 0.08 | 7.9 |
| Haemophilus parainfluenzae parC conferring resistance to fluoroquinolones | 2256 | 0.09 | 8.96 |
| Salmonella enterica gyrA conferring resistance to fluoroquinolones | 2637 | 0.03 | 2.66 |
| Capnocytophaga gingivalis gyrA conferring resistance to fluoroquinolones | 2511 | 0.02 | 2.11 |
| Salmonella enterica parC conferring resistance to fluoroquinolones | 2259 | 0.27 | 27.33 |
| Enterococcus faecium EF-Tu mutants conferring resistance to GE2270A | 1272 | 0.16 | 15.81 |
| Escherichia coli 16S rRNA mutation conferring resistance to edeine | 1542 | 0.31 | 29.14 |
| Salmonella enterica 16S rRNA (rrsD) mutation conferring resistance to spectinomycin | 1544 | 0.27 | 25.79 |
| Escherichia coli 16S rRNA (rrsH) mutation conferring resistance to spectinomycin | 1542 | 0.09 | 9.28 |
| Pasteurella multocida 16S rRNA mutation conferring resistance to spectinomycin | 1551 | 0.22 | 21.87 |
| Neisseria meningitidis 16S rRNA mutation conferring resistance to spectinomycin | 1544 | 0.06 | 6.03 |
| Propionibacterium acnes 16S rRNA mutation conferring resistance to tetracycline | 1486 | 0.04 | 3.7 |
| Moraxella catarrhalis 23S rRNA with mutation conferring resistance to macrolide antibiotics | 3156 | 0.61 | 54.45 |
| Escherichia coli 23S rRNA with mutation conferring resistance to clindamycin | 2904 | 0.14 | 14.09 |

**Table 2**. Molecular antibiotic resistance profile of the *A.baumanni* metagenomic sequences

| **Taxonomy** | **Mapped gene size** | **Avg depth** | **Coverage（%）** |
| --- | --- | --- | --- |
| OXA-371 | 826 | 0.06 | 5.94 |
| OXA-90 | 825 | 0.18 | 13.59 |
| QnrB66 | 645 | 0.13 | 6.83 |
| vanYA | 912 | 0.05 | 4.72 |
| TLA-2 | 915 | 0.13 | 4.49 |
| mdtF | 3115 | 0.04 | 1.51 |
| OXA-98 | 825 | 0.24 | 8.25 |
| adeF | 1219 | 6.49 | 94.58 |
| vgaALC | 1569 | 0.08 | 2.81 |
| OXA-376 | 826 | 0.48 | 7.76 |
| IMP-33 | 741 | 0.25 | 6.49 |
| IMP-51 | 742 | 0.17 | 5.8 |
| arlS | 1357 | 0.1 | 6.86 |
| adeS | 1086 | 9.19 | 97.6 |
| APH(3')-VIIa | 754 | 0.11 | 5.31 |
| APH(9)-Ia | 996 | 0.09 | 4.42 |
| OXA-208 | 825 | 0.06 | 6.07 |
| ErmQ | 775 | 0.18 | 5.94 |
| adeC | 1398 | 0.07 | 6.51 |
| vanL | 1051 | 0.09 | 4.38 |
| qacA | 1545 | 0.09 | 8.74 |
| mepA | 1356 | 0.07 | 3.32 |
| OXA-380 | 826 | 7.18 | 78.79 |
| OXA-377 | 826 | 0.48 | 15.27 |
| DHA-16 | 1140 | 0.04 | 4.04 |
| sav1866 | 1738 | 0.06 | 2.82 |
| dfrG | 499 | 0.09 | 9.04 |
| catB | 660 | 0.07 | 6.53 |
| rosB | 1693 | 0.02 | 2.42 |
| AcrS | 664 | 0.13 | 6.64 |
| OXA-386 | 826 | 0.36 | 21.33 |
| adeH | 1453 | 6.32 | 93.11 |
| TLA-1 | 945 | 0.04 | 4.34 |
| qacB | 1546 | 0.06 | 3.04 |
| OXA-136 | 811 | 0.06 | 5.56 |
| lmrB | 1434 | 0.03 | 3.07 |
| abeS | 331 | 7.96 | 94.24 |
| OXA-385 | 826 | 0.91 | 19.88 |
| vanHB | 972 | 0.09 | 4.43 |
| catB10 | 634 | 0.07 | 7.27 |
| adeJ | 3178 | 9.89 | 98.96 |
| tet(31) | 1233 | 0.07 | 3.57 |
| vanRE | 691 | 0.07 | 6.67 |
| Erm(42) | 906 | 0.05 | 4.86 |
| PC1 beta-lactamase (blaZ) | 847 | 0.2 | 13 |
| AAC(6')-Iae | 553 | 0.07 | 7.25 |
| CfxA | 967 | 0.05 | 4.55 |
| cmeB | 3124 | 0.01 | 1.41 |
| tetB(P) | 1960 | 0.02 | 2.25 |
| lnuB | 804 | 0.1 | 4.86 |
| cat | 649 | 0.54 | 20.68 |
| cat | 688 | 0.19 | 7.42 |
| cat | 649 | 0.14 | 13.58 |
| FosA3 | 418 | 0.11 | 11.03 |
| IND-14 | 721 | 0.13 | 6.39 |
| adeB | 3109 | 9.57 | 99.71 |
| OXA-383 | 826 | 0.91 | 17.58 |
| OXA-5 | 805 | 0.1 | 4.98 |
| lmrC | 1717 | 0.05 | 2.68 |
| AAC(6')-Ib-SK | 532 | 0.09 | 8.66 |
| vanZA | 486 | 0.28 | 12.78 |
| vgaD | 1579 | 0.03 | 2.92 |
| OXA-253 | 829 | 0.05 | 5.43 |
| IMI-7 | 879 | 0.26 | 10.59 |
| APH(3')-VIa | 781 | 0.05 | 5.38 |
| tet44 | 1924 | 0.02 | 2.13 |
| msrA | 1467 | 0.09 | 6.28 |
| adeG | 3181 | 5.22 | 79.75 |
| mel | 1465 | 0.06 | 2.94 |
| cmeR | 634 | 0.21 | 13.9 |
| OXA-192 | 810 | 0.11 | 5.81 |
| ErmT | 735 | 0.12 | 11.72 |
| Sed-1 | 888 | 0.05 | 4.74 |
| vanTE | 2110 | 0.04 | 2.09 |
| adeA | 1192 | 10.41 | 98.74 |
| mecA | 2008 | 0.02 | 2.19 |
| BcII | 772 | 0.06 | 6.1 |
| CcrA | 751 | 0.12 | 6.4 |
| mdsB | 3169 | 0.01 | 1.39 |
| oleB | 1710 | 0.03 | 2.63 |
| OXA-390 | 826 | 0.12 | 6.67 |
| tet(K) | 1381 | 0.06 | 6.3 |
| adeI | 1252 | 8.37 | 98.48 |
| dfrE | 463 | 0.2 | 20.13 |
| Erm(43) | 732 | 0.13 | 6.98 |
| norA | 1165 | 0.04 | 4.12 |
| FosB | 417 | 0.11 | 10.82 |
| mecB | 2025 | 0.04 | 2.57 |
| vanE | 1060 | 0.04 | 4.25 |
| adeK | 1456 | 7.55 | 91.62 |
| AAC(6')-It | 441 | 0.1 | 10.23 |
| vanHM | 982 | 0.09 | 4.38 |
| emrA | 1174 | 0.32 | 4.52 |
| vanF | 1033 | 0.05 | 4.65 |
| tet(43) | 1561 | 0.03 | 2.82 |
| ErmY | 735 | 0.12 | 6.13 |
| KHM-1 | 727 | 0.06 | 5.79 |
| AAC(6')-Ie-APH(2'')-Ia | 1441 | 0.22 | 9.79 |
| adeN | 654 | 9.36 | 99.23 |
| mdtE | 1159 | 0.04 | 3.8 |
| spd | 775 | 0.05 | 5.43 |
| mtrD | 3205 | 0.04 | 1.56 |
| tet(38) | 1353 | 0.07 | 3.25 |
| blt | 1204 | 0.04 | 3.91 |
| mefA | 1219 | 0.03 | 3.45 |
| vanA | 1032 | 0.04 | 4.27 |
| cat86 | 663 | 0.07 | 6.95 |
| patA | 1380 | 0.1 | 3.99 |
| patB | 1167 | 0.11 | 3.77 |
| vgaC | 231 | 0.39 | 21.3 |
| ESP-1 | 873 | 0.1 | 4.82 |
| vanSI | 1179 | 0.04 | 3.65 |
| mefC | 1224 | 0.11 | 3.52 |
| Listeria monocytogenes mprF | 2598 | 0.02 | 1.77 |
| Clostridium perfringens mprF | 1710 | 0.16 | 6.44 |
| Mrx | 1239 | 0.04 | 3.55 |
| cdeA | 1326 | 0.03 | 3.32 |
| mfd | 3447 | 0.03 | 1.42 |
| ADC-2 | 1152 | 8.66 | 88.01 |
| OXA-368 | 2721 | 0.02 | 1.51 |
| abcA | 4568 | 0.02 | 1.03 |
| farA | 1155 | 0.2 | 5.2 |
| hp1184 | 1380 | 0.03 | 3.19 |
| Erm(44) | 756 | 0.06 | 5.83 |
| tetA(60) | 1740 | 0.03 | 2.65 |
| adeL | 1014 | 10.38 | 99.9 |
| ANT(3'')-IIa | 689 | 13.07 | 99.71 |
| LpeB | 3045 | 0.04 | 2.89 |
| Klebsiella pneumoniae OmpK37 | 1463 | 0.06 | 3.01 |
| Bla2 | 771 | 0.05 | 5.45 |
| MCR-1.2 | 1626 | 0.08 | 2.58 |
| Moraxella catarrhalis M35 | 1077 | 0.13 | 4.28 |
| Staphylococcus aureus rpoB mutants conferring resistance to rifampicin | 3553 | 0.7 | 20.05 |
| Escherichia coli gyrB conferring resistance to aminocoumarin | 2416 | 0.26 | 10.68 |
| Staphylococcus aureus parE conferring resistance to fluoroquinolones | 1999 | 0.18 | 6.86 |
| Staphylococcus aureus pgsA mutations conferring resistance to daptomycin | 579 | 0.24 | 12.8 |
| Escherichia coli rpoB mutants conferring resistance to rifampicin | 4030 | 1.07 | 28.02 |
| Staphylococcus aureus rpoC conferring resistance to daptomycin | 3625 | 0.79 | 22.68 |
| Staphylococcus aureus mprF with mutation conferring resistance to daptomycin | 2511 | 0.03 | 3.23 |
| Staphylococcus aureus gyrA conferring resistance to fluoroquinolones | 2662 | 0.07 | 3.34 |
| Mycobacterium tuberculosis ndh with mutation conferring resistance to isoniazid | 1393 | 0.03 | 3.16 |
| Bartonella bacilliformis gyrB conferring resistance to aminocoumarin | 2431 | 0.16 | 5.76 |
| Staphylococcus aureus gyrB conferring resistance to aminocoumarin | 1933 | 0.07 | 4.55 |
| Clostridium difficile EF-Tu mutants conferring resistance to elfamycin | 1194 | 0.16 | 6.12 |
| Escherichia coli EF-Tu mutants conferring resistance to kirromycin | 1185 | 2.56 | 42.91 |
| Ureaplasma urealyticum gyrB conferring resistance to fluoroquinolone | 1953 | 0.1 | 5.23 |
| Escherichia coli EF-Tu mutants conferring resistance to Pulvomycin | 1230 | 0.04 | 3.74 |
| Escherichia coli gyrA conferring resistance to fluoroquinolones | 2629 | 0.08 | 3.54 |
| Escherichia coli parE conferring resistance to fluoroquinolones | 1894 | 0.13 | 6.34 |
| Morganella morganii gyrB conferring resistance to fluoroquinolone | 2416 | 0.31 | 6.13 |
| Mycobaterium leprae gyrA conferring resistance to fluoroquinolones | 3751 | 0.01 | 1.25 |
| Staphylococcus aureus parE conferring resistance to aminocoumarin | 1992 | 0.19 | 10.75 |
| Mycoplasma hominis parC conferring resistance to fluoroquinolone | 2802 | 0.03 | 3.18 |
| Salmonella enterica soxR with mutation conferring antibiotic resistance | 459 | 0.21 | 11.79 |
| LpxA | 789 | 8.55 | 99.49 |
| LpxC | 903 | 11.96 | 99.33 |
| Streptococcus pneumoniae parC conferring resistance to fluoroquinolone | 2473 | 0.07 | 3.68 |
| Haemophilus parainfluenzae gyrA conferring resistance to fluoroquinolones | 2646 | 0.18 | 7.03 |
| Haemophilus parainfluenzae parC conferring resistance to fluoroquinolones | 2256 | 0.19 | 7.27 |
| Salmonella enterica gyrA conferring resistance to fluoroquinolones | 2637 | 0.04 | 3.76 |
| Capnocytophaga gingivalis gyrA conferring resistance to fluoroquinolones | 2511 | 0.02 | 1.95 |
| Shigella flexneri gyrA conferring resistance to fluoroquinolones | 2628 | 0.05 | 1.9 |
| Enterococcus faecium EF-Tu mutants conferring resistance to GE2270A | 1272 | 1.27 | 30.06 |
| Ureaplasma urealyticum parC conferring resistance to fluoroquinolone | 2553 | 0.11 | 7.21 |
| Escherichia coli 16S rRNA mutation conferring resistance to edeine | 1542 | 2.21 | 38.48 |
| Mycobacterium tuberculosis 16S rRNA mutation conferring resistance to amikacin | 1537 | 0.03 | 3.19 |
| Salmonella enterica 16S rRNA (rrsD) mutation conferring resistance to spectinomycin | 1544 | 1.1 | 31.89 |
| Neisseria gonorrhoeae 16S rRNA mutation conferring resistance to spectinomycin | 1551 | 0.79 | 27.1 |
| Mycobacterium smegmatis 16S rRNA (rrsA) mutation conferring resistance to neomycin | 1529 | 0.03 | 3.14 |
| Mycobacterium chelonae 16S rRNA mutation conferring resistance to kanamycin A | 1441 | 0.23 | 8.12 |
| Escherichia coli 16S rRNA (rrsH) mutation conferring resistance to spectinomycin | 1542 | 0.53 | 6.94 |
| Mycobacterium abscessus 16S rRNA mutation conferring resistance to tobramycin | 1504 | 0.03 | 3.33 |
| Chlamydophila psittaci 16S rRNA mutation conferring resistance to spectinomycin | 1507 | 0.03 | 3.12 |
| Mycobacterium smegmatis 16S rRNA (rrsB) mutation conferring resistance to hygromycin B | 1454 | 0.06 | 2.75 |
| Pasteurella multocida 16S rRNA mutation conferring resistance to spectinomycin | 1551 | 2.57 | 24.32 |
| Borrelia burgdorferi 16S rRNA mutation conferring resistance to kanamycin | 1477 | 0.17 | 6.84 |
| Neisseria meningitidis 16S rRNA mutation conferring resistance to spectinomycin | 1544 | 0.56 | 11.28 |
| Propionibacterium acnes 16S rRNA mutation conferring resistance to tetracycline | 1486 | 0.03 | 3.37 |
| Helicobacter pylori 16S rRNA mutation conferring resistance to tetracycline | 1501 | 0.1 | 6.87 |
| Chlamydomonas reinhardtii 16S rRNA (rrnS) mutation conferring resistance to streptomycin | 1474 | 0.25 | 11.13 |
| Staphylococcus aureus 23S rRNA with mutation conferring resistance to linezolid | 2926 | 0.03 | 3.32 |
| Moraxella catarrhalis 23S rRNA with mutation conferring resistance to macrolide antibiotics | 3156 | 4.71 | 69.57 |
| Chlamydomonas reinhardtii 23S rRNA with mutation conferring resistance to erythromycin | 3403 | 0.01 | 1.44 |
| Helicobacter pylori 23S rRNA with mutation conferring resistance to clarithromycin | 2975 | 0.08 | 3.33 |
| Escherichia coli 23S rRNA with mutation conferring resistance to clindamycin | 2904 | 3.57 | 35.96 |
| Propionibacteria 23S rRNA with mutation conferring resistance to macrolide antibiotics | 3135 | 0.02 | 1.56 |
| Mycobacterium abscessus 23S rRNA with mutation conferring resistance to clarithromycin | 3112 | 0.01 | 1.41 |
| Mycobacterium avium 23S rRNA with mutation conferring resistance to clarithromycin | 3112 | 0.01 | 1.48 |
| Mycobacterium intracellulare 23S rRNA with mutation conferring resistance to clarithromycin | 3103 | 0.12 | 2.35 |
| Mycobacterium smegmatis 23S rRNA with mutation conferring resistance to clarithromycin | 3162 | 0.03 | 1.58 |
| Mycoplasma fermentans 23S rRNA with mutation conferring resistance to macrolide antibiotics | 2886 | 0.31 | 4.54 |
| Mycoplasma pneumoniae 23S rRNA mutation conferring resistance to erythromycin | 2905 | 0.11 | 4.1 |
| Brachyspira hyodysenteriae 23S rRNA with mutation conferring resistance to tylosin | 2996 | 0.02 | 1.64 |
